# Impacts of* FcγRIIB* and* FcγRIIIA* gene polymorphisms on systemic lupus erythematous disease activity index

**DOI:** 10.1186/s13104-021-05868-2

**Published:** 2021-12-18

**Authors:** Mansoor Karimifar, Khosro Akbari, Reza ArefNezhad, Farshid Fathi, Mohammad Moosaeepour, Hossein Motedayyen

**Affiliations:** 1 Department of Rheumatology, Alzahra Hospital, Isfahan University of Medical Sciences, Isfahan, Iran; 2 Department of Anatomy, School of Medicine, Shiraz University of Medical Sciences, Shiraz, Iran; 3 Department of Immunology, School of Medicine, Isfahan University of Medical Sciences, Isfahan, Iran; 4 Department of Community Medicine, School of Medicine, Isfahan University of Medical Sciences, Isfahan, Iran; 5 Autoimmune Diseases Research Center, Shahid Beheshti Hospital, Kashan University of Medical Sciences, 5th Kilometer of Ravand Road, Kashan, Iran

**Keywords:** Systemic lupus erythematous, Fcγ receptor IIB, Fcγ receptor IIIA, SLE disease activity index, Polymorphism

## Abstract

**Objective:**

Systemic lupus erythematous (SLE) disease is a chronic autoimmune disease with unknown etiology that can involve different organs. Polymorphisms in Fcγ receptors have been identified as genetic factors in susceptibility to SLE. This study was aimed to investigate effects of two single nucleotide polymorphisms (SNPs) within *FcγRIIB* and *FcγRIIIA* genes on systemic lupus erythematous disease activity index (SLEDAI) in an Iranian population.

**Results:**

Our findings indicated TT and GG genotypes were the common genotypes of FcγRIIB and FcγRIIIA SNPs in SLE patients, respectively. There were no significant differences in genotype and allele frequencies of FcγRIIB and FcγRIIIA SNPs in SLE and healthy subjects. However, the frequencies of genotypes and alleles of FcγRIIB and FcγRIIIA SNPs were significantly associated with some clinical manifestations used to determine SLEDAI (P < 0.001–0.5).

**Supplementary Information:**

The online version contains supplementary material available at 10.1186/s13104-021-05868-2.

## Introduction

Systemic lupus erythematosus (SLE) is a systemic autoimmune disease with unknown etiology described by the lack of immunological tolerance to self-antigens, atypical T-and B-cell reactions with the production of antibodies against self-antigens [1–4]. Genetic, environmental, and hormonal factors play important roles in disease susceptibility [5]. Genetic variations in the genes involved in immune functions contribute to the development of autoimmune disorders [1]. Thus, immune receptor genes such as *fragment crystallizable (Fc) receptor (FcR)* and *programmed cell death 1 (PDCD1)* have always been candidate genes in polymorphism studies [6, 7].

FcRs on the leukocytes bind to the FC region of antibodies and enhance some functions, including phagocytosis of antibody-antigen complexes and generation of signals regulating cell activities [8]. Fc gamma receptor (FCγR) plays an important role in phagocytosis of antigens opsonized with complement component. Based on its hybrid desire, it is divided into three groups (FcγRI or CD64, FcγRII or CD32, and FcγRIII or CD16), which each of them exerts different functions [9, 10]. The genes coding FcγRII (CD32) and FcγRIII (CD16) are located on chromosome 1q23.3 [11]. Several genome-wide screens have shown that single nucleotide polymorphism (SNP) as a genetic variation can impair and/or change the normal functions of FcγRs and thereby results in the development of autoimmunity such as rheumatoid arthritis and SLE [12]. Some reports have indicated that SLE patients suffered from the reduced expressions of FcγRII and FcγRIII [13]. Furthermore, it is reported that the reduced expression of FcγRIIIA has a protective effect on lupus-susceptible mice through inhibiting the progression of lupus nephritis [14]. FcγRIIB, unlike FcγRIIC and FcγRIIA, exerts immunosuppressive impacts on some immune cells such as monocytes and B cells. FcγRIIB binding to its ligand (FC region of IgG) produces an inhibitory signal leading to a reduction in cell activation [15]. Genetic variation in *FcγRII* gene may contribute to SLE susceptibility in some populations [16]. Until now, several studies have been performed to clarify possible effects of FcγR polymorphisms, especially type IIA, IIB, and IIIA, on susceptibility to autoimmune diseases in different populations. Some reports have revealed that these SNPs play indispensible roles in disease susceptibility and sickness period in autoimmune disorders such as SLE [17–21].

Regarding the fact that FcγR polymorphisms act as genetic risk factor in developing autoimmunity [22] and their roles in SLE development have not yet been identified in the Iranian population, this study was aimed to determine whether two SNPs (rs1050501 and rs396991) in *FcγRIIB* and *IIIA* genes may associate with systemic lupus erythematosus disease activity index (SLEDAI) in the Iranian population.

## Main text

### Methods

#### Study samples

The study populations comprised of 80 unrelated SLE patients and 95 sex-and age-matched healthy individuals without history of autoimmune disorders and cancers (Additional file 1: Table S1). The patients approved for SLE disease by the specialist based on the SLE American College of Rheumatology (ACR) classification criteria [23]. Patients were interviewed by the specialist and disease activity index (DAI) was provided by a questionnaire according to SLEDAI-2K (30 days) guideline. The questionnaire contained 24 items, including seizure, psychosis, organic brain syndrome, visual disturbance, cranial nerve disorder, lupus headache, cerebrovascular accident (CVA), vasculitis, arthritis, myositis, urinary casts, hematuria, proteinuria, pyuria, rash, alopecia, mucosal ulcer, pleurisy, pericarditis, low complement, increased DNA binding, fever, thrombocytopenia, and leukopenia. There were eight scores (range: 0 to 8) for answering each item depending on symptom severities. Patients with more than 6 score were considered active. The researchers were blinded to patient information and contributed to this study without any costs. Participants were Persian ethnic and chosen from Isfahan province of Iran. The study was confirmed by the Ethics Committee of Isfahan University of Medical Sciences (ethic code: Ir.mui.rct.1396.3.668) and carried out based on the Helsinki declaration. Written informed consent was obtained from all subjects before entering the study.

#### Assessment of SNPs in extracted DNA

EDTA-treated blood samples (5 ml) were collected from participants. Genomic DNA was extracted from the leukocytes using a QIAamp DNA Mini kit (Qia gene, Germany) according to the manufacturer’s instructions. The yield and purity of DNAs were determined by nano drop (BioTek, Epoch, USA) and quality of DNAs were evaluated by electrophoresis gel. Afterwards, two SNPs were investigated by real-time polymerase chain reaction with high resolution melting (real-time PCR-HRM) analysis. Each reaction for FcγIIB and IIIA SNPs were carried out in a 10 μl mixture (Additional file 2: Table S2). Real-time PCR-HRM was done using Rotor Gene 6000 machine (Qiagen, Hilden, Germany). The machine was programmed as described previously [24]. To determine SNPs, the melting curves were analyzed in the temperature range of 65 °C to 95 °C at the end of each run. All reactions were carried out in duplicate. Primer sequences were indicated in Additional file 3: Table S3.

#### Statistical analysis

Data were analyzed using SPSS (v18; SPSS Inc. Chicago, IL, USA). Chi‐square and Fisher's exact tests were used to evaluate the associations of genotype and allele frequencies with SLE susceptibility and SLEDAI. Bonferroni correction, as a post hoc analysis, was used to the pairwise comparisons with significance level of 0.017. P-value < 0.05 was statistically considered significant.

### Results

#### Description of patients

In this study, the most frequent clinical manifestations among 80 SLE patients were rash, arthritis, and leucopenia, while the most common laboratory manifestations were anti-nuclear antibodies (100%), anti-ds DNA (97.5%) and low complement (83.75%). The clinical and laboratory characteristics of patients with SLE are shown in Table 1.

**Table 1:** The clinical and laboratory characteristics of patients with SLE

The clinical and laboratory manifestations	Total (n = 80)
Seizure	10 (12.5%)
Psychosis	4 (5%)
Rash	66 (82.5%)
Organic Brain Syndrome	2 (2.5%)
Visual disturbance	2 (2.5%)
Cranial nerve disorder	3 (3.75%)
Lupus headache	4 (5%)
CVA	0 (0.0%)
Vasculitis	2 (2.5%)
Myositis	4 (5%)
Urinary casts	6 (7.5%)
Hematuria	6 (7.5%)
Proteinuria	8 (10%)
Pyuria	5 (6.25%)
Alopecia	2 (2.5%)
Pleurisy	4 (5%)
Precardia	7 (8.75%)
Low complement	59 (73.75%)
Increased DNA binding	25 (31.25%)
Fever	11 (13.755)
Thrombocytopenia	9 (11.25%)
Leukopenia	27 (26.25%)

#### Associations of genotype and allele frequencies of FcγRIIB and FcγRIIIA with SLE susceptibility

Our data revealed that there were no significant differences in FcγRIIB genotype frequencies between patient and control groups (Table 2). Allelic analysis indicated that C and T allele frequencies of FcγRIIB in patients did not significantly differ from those of control group (Table 2).

**Table 2:** The genotypes and allele frequencies of FcγRIIB (rs1050501) and FcγRIIIA (rs396991) SNPs in patient and control groups

Positions	Genotype and allele frequencies	Patients	Controls	OR (95% CI)	P value
		2n = 160	2n = 190		
Rs1050501	CC	25 (31.25%)	28 (29.47%)		0.64
	CT	30 (37.5%)	36 (37.89%)		
	TT	75 (46.8%)	31 (32.63%)		
	C	85 (53.2%)	92 (48.43%)	0.93 (0.61–1.43)	0.77
	T	38 (47.5%)	98 (51.57%)		
Rs396991	TT	25 (31.25%)	42 (44.21%)		0.73
	TG	17 (21.25%)	35 (36.84%)		
	GG	101 (63.1%)	18 (18.94%)		
	T	59 (36.9%)	119 (62.63%)	1.02 (0.66–1.57)	0.92
	G	25 (31.25%)	71 (37.36%)		

Values are indicated as count (percent)

The significance level was considered as P < 0.05

In addition, statistical analyses indicated that FcγRIIIA genotype frequencies were not significantly different from those of healthy individuals (Table 2). As shown in Table 2, no significant differences were observed in T and G allele frequencies between patient and control groups. Other results indicated that T allele was the most frequent allele of FcγRIIIA SNP (Table 2).

#### Correlations of FcγRIIB and FcγRIIIA with SLEDAI

To determine the associations of FcγRIIB and FcγRIIIA with SLEDAI, the relationships of genotype and allele frequencies of these SNPs with some clinical parameters, which were the most frequent clinical manifestations of SLE patients, were evaluated. Our data indicated that genotype and allele frequencies of FcγRIIB were significantly associated with the incidences of leucopenia, rash, mucosal ulcer, arthritis, thrombocytopenia in SLE patients (P < 0.001–0.05, Table 3). The results of multiple comparisons revealed that there were significant correlations between distribution frequencies of FcγRIIB CC, CT and TT genotypes versus (vs) other genotypes and these clinical characteristics used to determine SLEDAI (P < 0.001–0.05, Table 3).

**Table 3:** The correlations of rs1050501 and rs396991 with some clinical manifestations used to SLEDAI

Positions	Genotype and allele frequencies	Rash		P value	Thrombocytopenia		P value	Leukopenia		P value	Ulcer		P value	Arthritis		P value
		Yes	No		Yes	No		Yes	No		Yes	No		Yes	No	
Rs1050501	CC	7	18	0.001	16	9	< 0.001	14	11	0.001	16	9	0.001	6	19	0.001
	CT	8	17	0.001	19	6	< 0.001	17	8	0.001	16	9	0.001	9	16	0.001
	TT	14	16	0.08	21	9	< 0.001	20	10	0.001	19	11	0.001	12	18	0.001
	C	22	53	0.02	51	24	0.001	45	30	0.03	48	27	0.03	21	54	0.001
	T	36	49		61	24		57	28		54	31		33	52	
Rs396991	TT	11	27	0.001	25	13	0.01	17	21	0.41	22	16	0.65	12	26	0.01
	TG	9	16	0.02	13	12	0.65	15	10	0.021	17	8	0.02	13	12	0.42
	GG	6	8	0.001	9	8	0.72	8	9	0.71	10	7	0.03	9	8	0.51
	T	31	70	0.001	63	38	0.01	49	52	0.08	61	40	0.051	37	64	0.03
	G	21	32		51	28		32	28		37	22		31	28	

Furthermore, other results of statistical analyses showed that the frequencies of some genotype and allele of FcγRIIIA played significant roles in determining SLEDAI. Similar to FcγRIIB, our results indicated that some genotype and allele frequencies of FcγRIIIA were significantly correlated to SLEDAI (P < 0.001–0.05, Table 5). The frequencies of FcγRIIIA TT, TG and GG genotypes vs other genotypes were significantly associated with SLEDAI (P < 0.001–0.05, Table 3).

## Discussion

Numerous investigations stated that defects in genes related to the immune system can participate in the pathogenesis of autoimmune diseases such as SLE, but the roles of genetic agents have not clearly reported so far [25]. Thus, this study investigated rs1050501 and rs396991 SNPs and their associations with SLEDAI in an Iranian population from Isfahan province.

FcγRs have key roles in the immune system through regulating antibody effector functions. FcγRIIB expresses on different immune cells such as macrophages, B cells, granulocytes, and dendritic cells (DCs) [26]. This receptor suppresses the stimulation of B cells and reduces the development of autoimmunity. Some reports have shown that FcγRIIB SNP results in an amino acid substitution of threonine for isoleucine at position 232 (T232I), leading to decreased suppressor activities and thereby enhances susceptibility to SLE [27].

In this case–control study, genotype frequencies of FcγRIIB SNP showed that TT genotype frequency was higher in patient than healthy group, although the difference was not statistically significant. In agreement with this finding, Jeon et al. reported that a tendency for increase of TT genotype in SLE patients compared with healthy individuals, however, no significant difference was observed in this genotype between patient and healthy groups [9]. This finding proposes a key question why there is an inconsistency between TT genotype and T allele frequency in patients with SLE. As mentioned previous, T allele frequency showed a reduction in patients compared with healthy subjects. Despite a tendency for increase of CC genotype in SLE patients, the differences in CC and CT genotype frequencies between patient and healthy groups were not statistically significant. The increased frequency of CC genotype in patients was consistent with the tendency for increase of C allele number. Our data revealed that although there were some changes in the frequencies of genotypes and alleles between SLE patients and healthy subjects, these differences were not statistically considerable and rs1050501 was not associated with SLE susceptibility. In contrast with these observations, Zhu et al. in a meta-analysis study concluded that C allele of rs1050501 could be effective in SLE susceptibility and disease progression [28]. Furthermore, another study conducted by Pan et al. on 119 SLE patients from 95 nuclear families has indicated that C and T alleles of FcγRIIB were significantly associated with SLE and CT genotype was the most frequent genotype of FcγRIIB in SLE patients [29]. Although rs1050501 failed to show impact on SLE susceptibility in our study population, other statistical analyses indicated that genotype and allele frequencies of FcγRIIB could be considered as genetic factors to clarify SLEDAI in Iranian SLE patients. We observed that the frequencies of FcγRIIB genotypes and alleles were significantly correlated to some clinical parameters used to determine SLEDAI. Similar to this finding, a published short report manifested that the polymorphism of homozygous FcγRIIB-I232 was dramatically related to the elevated SLEDAI score [30]. Another study on other genotypes and alleles of FcγRIIB in an Indian population has revealed that the T allele was correlated to severity and clinical parameters of SLE [18].

*FcγRIIIA* gene is located on chromosome 1q23.3 and encodes for FcγRIIIA, a glycosylated heterogeneous form of FcγR [31, 32]. This receptor binds to immune complexes with low tendency and interestingly interacts with IgG2 subclass [33, 34]. It is stated that differences in genotypes and alleles of FcγRIIIA may be associated with autoimmunity [35]. Other results of this study indicated that GG genotype of rs396991 polymorphism had higher frequency in patients than healthy subjects. However, this difference was not statistically significant. On the contrary, TT, GT genotype frequencies were lower in patients than control group. These findings were agreed with the increased frequency of T allele in healthy subjects compared with SLE patients. Our data demonstrated that the frequency of G allele was in contrast with GG genotype frequency of FcγRIIIA SNP. Similar to the data of FcγRIIB SNP, allele and genotype frequencies of FcγRIIIA SNP in patients were not significant different from those of control group. These observations are consistent with the results of a study conducted on SLE patients showing the differences in genotypes and alleles of FcγRIIIA SNP between SLE and healthy subjects were not statistically significant [36]. Alansari et al. conducted a study on five SNPs in *FcγR* gene in three ethnic groups of SLE patients. The authors reported that genotype and allele frequencies of five SNPs in patients with SLE did not statistically differ from those of healthy subjects and subsequently concluded FcγR SNPs could not contribute to SLE susceptibility [37]. However, there are some studies revealing FcγRIIB and FcγRIIIA SNPs may participate in SLE development [38]. It is shown that 232TT genotype had an increased frequency in SLE patients compared with healthy subjects [39]. Dhaouadi et al. stated significant differences in allele and genotype frequencies of FcγRIIIA SNP between SLE and normal subjects in a Tunisian population [40]. In addition, other studies have reported that the decreased expression of FcγRIIIA contributed to SLE severity, but no significant relationship was observed between FcγRIIIA expression level and SLEDAI (38). In contrast, we found that the frequencies of some genotypes and alleles of FcγRIIIA were significantly associated with SLEDAI. This discrepancy may attribute to ethnic and geographic differences, inter-study heterogeneity in the studied SNPs and alleles, and sample size used in different studies.

Overall, although the current study failed to show the impacts of FcγRIIB and FcγRIIIA SNPs on SLE susceptibility, our data for the first time indicate that FcγRIIB and FcγRIIIA SNPs are associated with SLEDAI in the Iranian population.

## Limitation

It should be noted that sample size may be a limitation of the study which may correlate to no correlations of these SNPs with SLE susceptibility. Therefore, more robust studies in different populations with larger sample size are needed to support our data and determine the effects of other *FcγR* SNPs in SLE development.

## Supplementary Information

**Additional file 1. ** Table. S1) Demographic characteristics of participants.

**Additional file 2.** Table S2) Components and their volumes used to PCR-HRM.

**Additional file 3.** Table. S3) Primer sequences used to PCR-HRM.

## Data Availability

The datasets generated and/or analysed during the current study are available in the ClinVar repository, Accession Numbers VCV000005467.1–VCV000225994.2.

## Abbreviations

SLE: Systemic lupus erythematosus
SNPs: Single nucleotide polymorphisms
SLEDAI: Systemic lupus erythematous disease activity index
FcγR: Fragment crystallizable (Fc) receptor
PDCD1: Programmed cell death 1
ACR: American College of Rheumatology
CVA: Cerebrovascular accident
DC: Dendritic cell
