# Correction to: Impacts of FcγRIIB and FcγRIIIA gene polymorphisms on systemic lupus erythematous disease activity index

**DOI:** 10.1186/s13104-022-06109-w

**Published:** 2022-07-14

**Authors:** Mansoor Karimifar, Khosro Akbari, Reza ArefNezhad, Farshid Fathi, Mohammad Mousaei Ghasroldasht, Hossein Motedayyen

**Affiliations:** 1 Department of Rheumatology, Alzahra Hospital, Isfahan University of Medical Sciences, Isfahan, Iran; 2 Department of Anatomy, School of Medicine, Shiraz University of Medical Sciences, Shiraz, Iran; 3 Department of Immunology, School of Medicine, Isfahan University of Medical Sciences, Isfahan, Iran; 4 Ariagene Medical Genetic Laboratory, Isfahan, Iran; 5 Department of Obstetrics and Gynecology, University of Chicago, 5841 S. Maryland Ave., Chicago, IL 60637 USA; 6 Autoimmune Diseases Research Center, Shahid Beheshti Hospital, Kashan University of Medical Sciences, 5th Kilometer of Ravand Road, Kashan, Iran

## Correction to: BMC Research Notes (2021) 14:455 10.1186/s13104-021-05868-2

Following the publication of the original article [1] the authors informed us that an incorrect author was inadvertently included in the author list.

The fifth author’s name “Mohammad Moosaeepour” should be replaced with that of the correct author, Dr. Mohammad Mousaei Ghasroldasht.

The author’s affiliation (affiliation 4) should also be corrected to:

4. Ariagene Medical Genetic Laboratory, Isfahan, Iran;

5. Department of Obstetrics and Gynecology, University of Chicago, 5841 S. Maryland Ave., Chicago, IL 60637, USA.

Finally, the “Authors’ contributions” section should be updated to:

“MK and KHA participated in the disease diagnosis, sample collections, and obtained funding for the work. FF and RA participated in the design of some experiments and statistical analysis of the data. MMG carried out some of the experiments. HM participated in the study design and drafted the manuscript. All authors read and approved the final manuscript.”

The correct author information has been included in the author list, reference and “Author details” section of this Correction.

The original article has been revised.

## References

[1] Karimifar M, Akbari K, ArefNezhad R, Fathi F, Ghasroldasht MM, Motedayyen H (2021). Impacts of FcγRIIB and FcγRIIIA gene polymorphisms on systemic lupus erythematous disease activity index. BMC Res Notes.

