# Supplementary figures and images for: Impacts of FcγRIIB and FcγRIIIA gene polymorphisms on systemic lupus erythematous disease activity index
Source: BMC Res Notes. 2021 Dec 18;14:455. doi: 10.1186/s13104-021-05868-2 (PMC8684074; doi:10.1186/s13104-021-05868-2)

**Table. S1) Demographic characteristics of participants**

|  |  | **Patient (n=80)** | **Control (n=95)** | |
| --- | --- | --- | --- | --- |
| **Age** | | 36 ± 9.8 | 37 | ± 10.11 |
|  | |  |  |  |
| **Sex** | Female | 72 (90%) | 83 | (87.36%) |
|  | Male | 8 (10%) | 12 | (12.64%) |
|  |  |  |  |  |


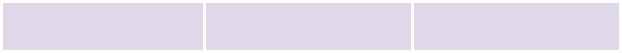

Supplement: Supplementary file 1 — Additional file 1. Table. S1) Demographic characteristics of participants. [file 13104_2021_5868_MOESM1_ESM.docx]
