# Supplementary material for: Impacts of FcγRIIB and FcγRIIIA gene polymorphisms on systemic lupus erythematous disease activity index
Source: BMC Res Notes. 2021 Dec 18;14:455. doi: 10.1186/s13104-021-05868-2 (PMC8684074; doi:10.1186/s13104-021-05868-2)
Supplement: Supplementary file 2 — Additional file 2. Table S2) Components and their volumes used to PCR-HRM. [file 13104_2021_5868_MOESM2_ESM.docx]

**Table S2) Components and their volumes used to PCR-HRM**

| **Components** | **Volumes** |
| --- | --- |
| Extracted DNA | 1μl (50 ng) |
| Eva Green | 2 μl |
| Forward primer | 0.5 μl (10 p.m) |
| Reverse primer | 0.5 μl (10 p.m) |
| MgCl2 | 1μl (50mM), |
| dNTP | 0.5 μl (10mM) |
| PCR buffer (10X) | 1.5 μl |
| Nuclease-free water | 2μl |
| Taq DNA polymerase | 1 μl (1U/µl) |
