# Supplementary material for: Impacts of FcγRIIB and FcγRIIIA gene polymorphisms on systemic lupus erythematous disease activity index
Source: BMC Res Notes. 2021 Dec 18;14:455. doi: 10.1186/s13104-021-05868-2 (PMC8684074; doi:10.1186/s13104-021-05868-2)
Supplement: Supplementary file 3 — Additional file 3. Table. S3) Primer sequences used to PCR-HRM. [file 13104_2021_5868_MOESM3_ESM.docx]

**Table. S3) Primer sequences used to PCR-HRM**

| **Positions** | **Primer sequences** |
| --- | --- |
| **Rs1050501** | Forward (5′–3′)  CTCCCCGTCTCTTCACCGATG  Reverse (5–3′)  TCAAGGCCACTACAGCAGC |
| **Rs396991** | Forward (5′–3′)  CCTTGAGTGATGGTGATGTTCA  Reverse (5′–3′)  CCAAAAGCCACACTCAAAGAC |
